# Network Meta-analysis of Randomized Trials on the Safety of Vascular Closure Devices for Femoral Arterial Puncture Site Haemostasis

**DOI:** 10.1038/srep13761

**Published:** 2015-09-08

**Authors:** Jun Jiang, Junjie Zou, Hao Ma, Yuanyong Jiao, Hongyu Yang, Xiwei Zhang, Yi Miao

**Affiliations:** 1 Department of General Surgery, the First Affiliated Hospital of Nanjing Medical University, No.300 Guangzhou Road, Nanjing 210029, China

## Abstract

The safety of vascular closure devices (VCDs) is still debated. The emergence of more related randomized controlled trials (RCTs) and newer VCDs makes it necessary to further evaluate the safety of VCDs. Relevant RCTs were identified by searching PubMed, EMBASE, Google Scholar and the Cochrane Central Register of Controlled Trials electronic databases updated in December 2014. Traditional and network meta-analyses were conducted to evaluate the rate of combined adverse vascular events (CAVEs) and haematomas by calculating the risk ratios and 95% confidence intervals. Forty RCTs including 16868 patients were included. Traditional meta-analysis demonstrated that there was no significant difference in the rate of CAVEs between all the VCDs and manual compression (MC). Subgroup analysis showed that FemoSeal and VCDs reported after the year 2005 reduced CAVEs. Moreover, the use of VCDs reduced the risk of haematomas compared with MC. Network meta-analysis showed that AngioSeal, which might be the best VCD among all the included VCDs, was associated with reduced rates of both CAVE and haematomas compared with MC. In conclusion, the use of VCDs is associated with a decreased risk of haematomas, and FemoSeal and AngioSeal appears to be better than MC for reducing the rate of CAVEs.

Manual compression (MC) is traditionally used to achieve haemostasis after coronary and peripheral angiography or angioplasty via the femoral artery. From the early 1990s, a variety of vascular closure devices (VCDs) have been developed to shorten the time-to-haemostasis and the time-to-ambulation^1^,^2^. These VCDs are mainly categorized into three different categories based on their mechanism of action, namely collagen plug-based VCDs, clips-based VCDs and suture -based VCDs. These devices may also reduce the risk of access site complications. However, the safety of VCDs has not yet been clarified by many studies; in particular, it has been covered by very few meta-analyses^1^,^2^,^3^,^4^,^5^. Moreover, the results of these studies are contradictory.

Recently, several more prospective, randomized trials have evaluated the efficacy and safety of VCDs^6^,^7^,^8^. In addition, new generations of various VCDs have been designed and applied clinically^7^. Therefore, it is necessary to review the efficiency and safety of VCDs in light of these new developments. To further examine the safety of VCDs with the up-to-date evidence, we conducted a meta-analysis of the studies so far in order to come to a more reliable conclusion. The network meta-analysis we performed here allows for the integration of data from direct and indirect comparisons of the safety of different VCDs.

## Materials and Methods

This systematic review was performed in accordance with the Preferred Reporting Items for Systematic Reviews and Meta-Analyses guidelines for meta-analyses of intervention trials^9^. The PROSPERO registration number is CRD42015015780.

### Information sources and search

PubMed, EMBASE, Google Scholar and the Cochrane Central Register of Controlled Trials were searched using the key words “vascular closure”, “arterial closure”, “arteriotomy closure”, “haemostasis”, and “manual compression” in December 2014. The following search strategy used in the PubMed database: ((((randomized controlled trial[pt] OR controlled clinical trial[pt] OR randomized[tiab] OR hemostasis[tiab] OR haemostasis[tiab] OR manual compression[tiab] OR clinical trials as topic[mesh:noexp] OR randomly[tiab] OR trial[ti]) NOT (animals[mh] NOT humans[mh])))) AND (((vascular closure devices[Title/Abstract]) OR vascular closure device[Title/Abstract]) OR closure devices[Title/Abstract]). References to previous meta-analyses and reviews were further manually searched. We will contact the original investigators for any missing data, if required.

### Eligibility criteria

Published prospective randomized controlled trials (RCTs) that compared different VCDs with MC and/or VCDs in patients undergoing any type of angiography or angioplasty via the femoral artery were included without any language restrictions. RCTs evaluating MC devices, such as haemostasis pads, sandbag, FemoStop, D-Stat Dry or C-Clamp versus MC or only one VCD were excluded. Case-control studies, cohort studies, case series, non-random designed trials and trials without the outcomes of interest or enough information for data extraction were excluded.

### Data collection

Two reviewers (Jun Jiang and Yuanyong Jiao) reviewed and extracted the data independently. Any disagreements between these two reviewers were resolved by a third reviewer (Xiwei Zhang). To assess the methodological quality of the included trials, we used the criteria for quality assessment recommended by the Cochrane Collaboration Handbook. The following items from each eligible study were included: the first author, year of publication, types of VCDs, sample size, number of combined adverse vascular events (CAVEs) and haematomas regardless of the size of each study arm, country, funding source, duration of follow-up and methodological aspects of each trial (Jadad score).

### Outcome measures

The outcomes assessed in this meta-analysis are the rates of CAVEs and all groin haematomas. CAVEs include access site complications such as major complications, including mortality; femoral artery complications requiring surgical vascular repair or blood transfusion; and minor complications including bleeding, groin haematoma, retroperitoneal haematoma, arteriovenous fistula, pseudoaneurysm, arterial dissection, limb ischaemia or distal embolization, local infection, deep vein thrombosis and femoral artery thrombosis. Groin pain, prolonged hospitalization, vagal episode reaction, oozing and prolonged bleeding requiring prolonged bed rest, adjunctive MC or no intervention were not considered as CAVE. Repetitive records of complications were screened and excluded. All groin haematomas detected by physical examination or ultrasound were recorded irrespective of the haematoma grade.

### Statistical analysis

The meta-analysis was performed using Review Manager 5.3 (Copenhagen: The Nordic Cochrane Centre, The Cochrane Collaboration, 2014). The risk for CAVE was expressed as the risk ratio (RR) with 95% confidence interval (CI). Heterogeneity was assessed by using I^2^, which was considered to be significant when its value was more than 50%. Data were pooled using the Mantel-Haenzel (M-H) fixed or random-effects model. The publication bias was assessed using a funnel plot. Moreover, Begg’s test was performed to detect the publication bias using STATA (version 10.1, StataCorp LP, College Station, TX). *P* < 0.05 (two-sided) was considered to indicate statistical significance. Network meta-analyses were conducted using the ADDIS software 1.16.5^10^. Network meta-analysis allows for the integration of data from direct and indirect comparisons and estimation of the effect of all the included treatments of all the included studies. Node-splitting analysis was used to assess whether direct and indirect evidence on a specific node were in agreement. In addition, the rank probability plot produced by the network meta-analysis was used to estimate the probability of each of the treatments being the best, the second best, etc^10^.

## Results

### Search results and study selection

**Figure 1:**
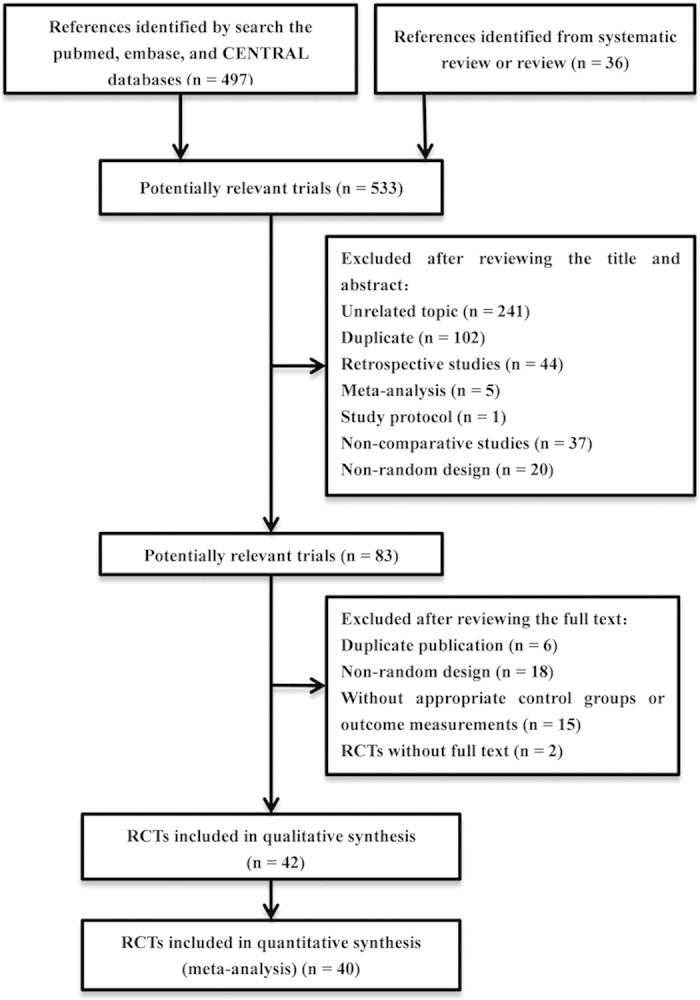
Study flow diagram showing the selection of articles for the meta-analysis. CENTRAL =  Cochrane Central Register of Controlled Trials, RCT = randomized controlled trial.

The search generated 533 citations. Four hundred and fifty citations were excluded after screening the titles and abstracts. After reading the full text, 41 citations were excluded. The reasons for exclusion were mainly duplicate publication, unrelated topics, retrospective studies, meta-analysis, non-comparative or non-random design studies, and studies without appropriate control groups or outcome measurements. In addition, two eligible trials^8^,^11^ for which the full text was not available were excluded. Two trials^12^,^13^ were further excluded due to zero events in both arms. Finally, 40 trials^6^,^7^,^14^,^15^,^16^,^17^,^18^,^19^,^20^,^21^,^22^,^23^,^24^,^25^,^26^,^27^,^28^,^29^,^30^,^31^,^32^,^33^,^34^,^35^,^36^,^37^,^38^,^39^,^40^,^41^,^42^,^43^,^44^,^45^,^46^,^47^,^48^,^49^,^50^,^51^ were included in this meta-analysis. The screening process is shown in Fig. 1.

### Characteristics of the included trials

General information about the included trials is shown in Supplementary Table S1. The trials included 38 English papers, 1 Spanish paper^29^ and 1 Chinese paper^49^. The sample size ranged from 22 to 1509, with a total size of 16868; the patients underwent coronary angiography, angioplasty and peripheral interventional procedures. Most patients in these studies underwent periprocedural anticoagulation or anti-platelet therapy, or both. Five studies compared two or more VCDs with MC^7^,^40^,^45^,^47^,^49^. Four studies compared one VCD with one or two VCDs^31^,^33^,^44^,^46^. The remaining 31 studies compared one VCD with MC. The risk of bias is shown in Supplementary Figure S1. In all the studies, blinding of the personnel and patients to treatment allocation was not feasible. Only four studies^7^,^16^,^18^,^44^ mentioned blinding of the outcome assessors in their study. Information on random sequence generation was adequate in 10 studies^6^,^7^,^14^,^18^,^19^,^38^,^40^,^44^,^48^,^50^, among which allocation concealment was adequate in 7 studies^6^,^7^,^18^,^19^,^38^,^44^,^48^. The follow-up period ranged from overnight to 1.11 years. Not every outcome of CAVE was examined in all the studies. There was no obvious publication bias in the studies based on the findings from the funnel plot (Supplementary Fig. S2) and Begg’s test (t = 0.35; *P* = 0.725).

### Direct comparison meta-analysis

Thirty-six studies including 15252 patients were included in the direct comparison meta-analysis. The VCDs examined were AngioSeal, VasoSeal, QuickSeal, ExoSeal, FemoSeal, Duett, Perclose (Prostar/Techstar/Proglide), EVS, StarClose, Boomerang and Bio-DISC were included (Supplementary Table S1). The other types of VCDs (Boomerang, EVS, Bio-DISC and Duett) were included in only one trial each, which was inadequate for the traditional meta-analysis. Comparison of different VCDs including AngioSeal, Perclose, VasoSeal, StarClose, ExoSeal, QuickSeal and FemoSeal with MC was conducted in this meta-analysis (Table 1).

When comparing any VCD with MC by traditional meta-analysis, the risk for CAVE seemed to be similar between the VCDs and MC (Heterogeneity: Chi^2^ = 108.07, I^2^ = 68%; test for overall effect: Z = 1.19, *P* = 0.23). When traditional meta-analysis was performed in eighteen studies with a Jadad score of 3, there was no difference in the risk for CAVE between the VCDs and MC (Heterogeneity: Chi^2^ = 64.94, I^2^ = 74%; test for overall effect: Z = 1.46, *P* = 0.15).

To exclude the potential bias induced by different populations, we excluded two studies^27^,^49^ conducted in the East Asian population; however, the risk for CAVE was still similar between the VCDs and MC (Heterogeneity: Chi^2^ = 101.88, I^2^ = 68%; test for overall effect: Z = 0.79, *P* = 0.43). To further reduce language bias, we excluded two non-English language studies^29^,^49^. The risk for CAVE was also similar between the VCDs and MC (Heterogeneity: Chi^2^ = 102.77, I^2^ = 68%; test for overall effect: Z = 0.98, *P* = 0.33).

**Figure 2:**
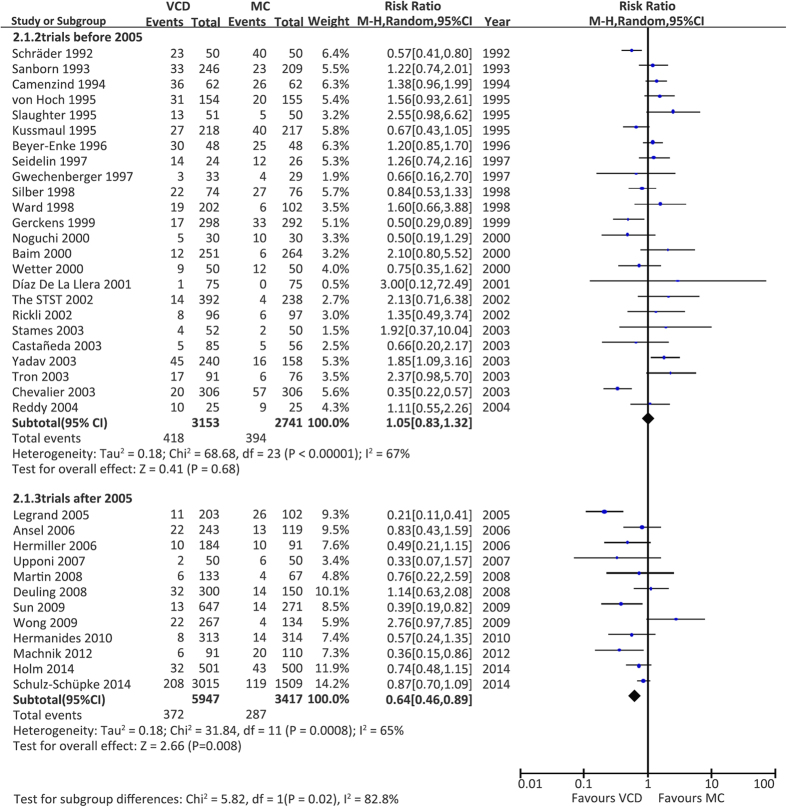
Subgroup analysis stratified by the year of publication accessing the risk of combined adverse vascular events of VCDs versus MC. VCD = vascular closure device, MC = manual compression, M-H = Mantel-Haenzel, CI = confidence interval.

Subgroup analysis of all the different types of VCDs except FemoSeal, which was associated with a significantly reduced risk of CAVE (Random effects, RR: 0.75, CI: 0.60–0.94, *P* = 0.01), showed similar results (Table 1). Taking into account the technical and design improvements of VCDs and increase in operator experience in the past decade, the application of VCDs after 2005 was associated with a decreased risk of VCD-associated complications. Subgroup analysis stratified by the year of publication revealed a trend toward decreased risk of CAVE in trials published after 2005 (Fig. 2). Similar results were found when we excluded two studies^27^,^49^ conducted in the East Asian population (Supplementary Fig. S3) or two non-English language studies^29^,^49^ (Supplementary Fig. S4), respectively, to explore the potential bias resulted from different populations and languages.

**Figure 3:**
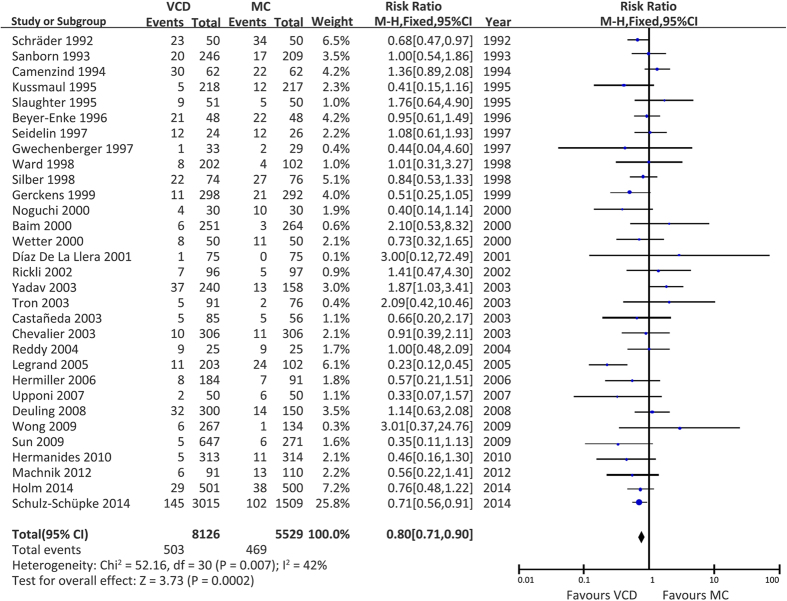
Risk of haematomas associated with all VCDs versus MC. VCD = vascular closure device, MC = manual compression, M-H = Mantel-Haenzel, CI = confidence interval.

We separately investigated the haematoma rate of VCDs versus MC. As some studies did not have enough information, only 31 studies including 13649 patients were included in this subgroup analysis. The results demonstrated that the haematoma risk was significantly lower in the VCD group than in the MC group (Fig. 3). Similar results were also detected when we excluded two studies^27^,^49^ conducted in the East Asian population (Supplementary Fig. S5) or two non-English language studies^29^,^49^ (Supplementary Fig. S6), respectively.

### Network meta-analysis

**Figure 4:**
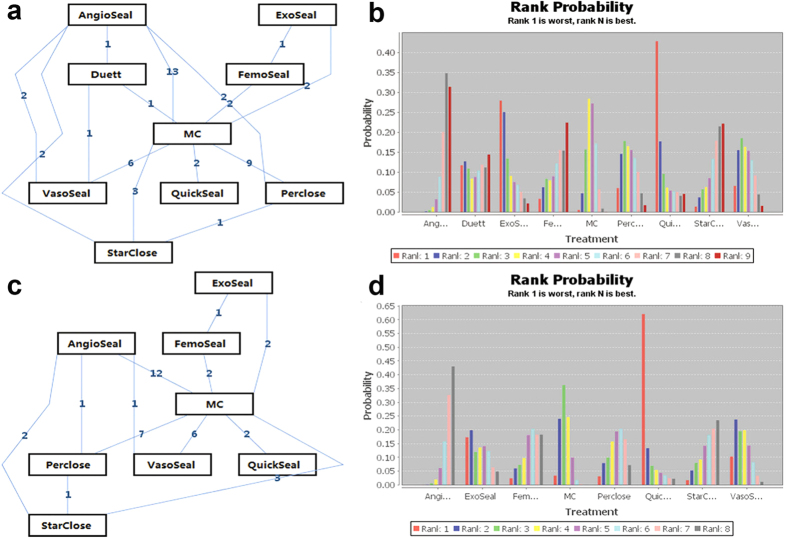
Network of the included vascular closure devices and rank probability plot derived from the network meta-analysis with respect to the risk for femoral artery puncture-related combined adverse vascular events (a,b) and hematomas (c,d). The figures in the lines of the network graph represent the number of direct comparisons between each pair of treatments. The rank probability plot produced by the network meta-analysis estimates the probability of each treatment being the best, the second best, etc.

Forty studies including 16051 patients were included in this network meta-analysis. Eight types of VCDs—AngioSeal, VasoSeal, QuickSeal, ExoSeal, FemoSeal, Perclose (Prostar/Techstar/Proglide), Duett and StarClose—were included for evaluating the CAVE risk (Fig. 4a). Node-splitting analysis showed that there were no significant differences between the direct and indirect comparisons (Supplementary Table S2). Therefore, a consistency model was used to evaluate the relative effect of the included VCDs and MC (Random effects, RR: 0.54, CI: 0.33–0.82). The relative effects of the included VCDs and MC were identical when we excluded two studies^27^,^49^ conducted in the East Asian population (Random effects, RR: 0.55, CI: 0.33–0.83) or two non-English language studies^29^,^49^ (Random effects, RR: 0.55, CI: 0.33–0.83), respectively. The current network meta-analysis showed that AngioSeal reduced the risk of CAVE compared with MC (Random effects, RR: 0.67, CI: 0.46–0.98). The other VCDs were associated with a similar risk for CAVE in comparison with MC. Moreover, there were no significant differences with regard to the risk for CAVE between these VCDs (Table 2). The rank probability plot indicated that AngioSeal might be the best VCD (Fig. 4b).

Separate network analysis for the risk of haematomas associated with the VCDs was investigated. Only 34 studies were included and 6 studies were excluded due to inadequate information. AngioSeal, VasoSeal, QuickSeal, ExoSeal, FemoSeal, Perclose (Prostar/Techstar/Proglide) and StarClose were examined (Fig. 4c). The results of the consistency model showed that AngioSeal, which reduced the risk of haematomas compared with MC (Random effects, RR: 0.57, CI: 0.39–0.85) (Table 3), might be the best VCD (Fig. 4d) of all the seven VCDs examined. The results of network meta-analysis for the risk of hematomas were also similar after we excluded the studies^27^,^49^ conducted in the in the East Asian population (Random effects, RR: 0.45, CI: 0.12–0.80; Supplementary Table S3) or two non-English language studies^29^,^49^ (Random effects, RR: 0.45, CI: 0.15–0.81; Supplementary Table S4), respectively.

## Discussion

Our results showed that the risk for CAVE was similar between all the included VCDs and MC. However, the results of meta-analysis of the trials published in the past decade revealed that the use of VCDs reduced the rate of CAVEs. Moreover, the results of subgroup analysis demonstrated that the use of VCDs was associated with a significant reduction in the rate of haematomas. Network meta-analysis suggested that AngioSeal, which reduced the rate of CAVEs and haematomas, might be the best VCD among the VCDs included in this study.

Due to the heterogeneity of the studies, including the ethnic group, diagnostic or interventional procedures, different anticoagulation or anti-platelet status of the patients, sheath size and so on, our comparative findings between VCDs and MC should be interpreted with caution. Only RCTs were included in our meta-analysis; however, the methodological quality of these RCTs varied. Most RCTs did not provide enough information on random sequence generation and allocation concealment. The differences in the duration of follow-up might lead to incomplete outcome data. Generally, more recently reported RCTs have relatively better quality. For example, the large-scale RCT by Schulz-Schupke *et al.*^7^ had adequate randomization, allocation concealment and blinding of outcome assessment. In addition, financial support from industries might guide researchers to focus on specific VCDs, thus providing enough data for the meta-analysis to reach a conclusion about these VCDs in particular. Nonetheless, the overall assessment showed that VCDs failed to reduce the rate of CAVEs. The results of subgroup analysis stratified by the year of publication showed that the pooled rate of CAVEs in the RCTs published after 2005 significantly decreased in the VCD group. Newer VCDs with improvements in device design and increase in the experience of the operators in the past decade might be one of potential reasons for this finding^52^, which favors the use of VCDs. In particular, meta-analysis of two trials with a large sample size demonstrated that FemoSeal, which is somewhat similar to Angio-Seal with regard to its mechanism of action, significantly reduced the incidence of access site CAVEs compared with MC. Moreover, the current network meta-analysis suggested that AngioSeal might be the best VCD among the VCDs included in the study. Therefore, it is reasonable to speculate that VCDs with a novel design may continue to be effective in decreasing the complication rate.

With regard to the total complication rate, our results were different from those of Nikolsky^4^ and Koreny^1^, which favored manual or mechanical compression. Our findings demonstrated that the use of VCDs significantly reduced the risk of haematomas. However, there are differences between our meta-analyses and the previous ones with regard to the quality of the included studies. Vaitkus *et al.*^3^, Koreny *et al.*^1^ and Biancari *et al.*^2^ all included RCTs in their meta-analyses, in which the control group was manual or/and mechanical compression. Nikolsky *et al.*^4^ included both RCTs and observational studies. Das *et al.*^5^ included both non-comparative and comparative studies. The pooled access-site-related complication rate also varied in these meta-analyses. Further, the total complication rate or incidence of groin haematoma, pseudoaneurysm, infection and lower limb ischaemia were examined in these meta-analyses. These discrepancies may account for the differences in the results or conclusions among the studies.

In this study, one limitation was that we used the rate of CAVE and haematomas regardless of their size as the primary end points. Various end points were measured including major and minor vascular complications across studies. For example, haematomas were detected by physical examination or ultrasonography, and defined by various grading criteria (mainly by the size of the diameter)^4^. These discrepancies may have resulted in inaccurate assessment of the pooled haematoma rate. In addition, bleeding, haematoma, retroperitoneal haematoma and femoral pseudoaneurysm may represent different stages of haemorrhagic complications and lead to repetitive records. The incidence of some outcome measures such as groin infection, distal embolism, and femoral artery or vein thrombosis was relatively low, especially in studies with a small sample size. It is also true that minor complications occur more frequently than major complications, and any complication would impair patient satisfaction and increase cost. Thus, it seems reasonable to use CAVE and the rate of all haematomas instead of the rate of all major complications as outcome measures to evaluate the safety of VCDs.

In conclusion, the results of the current traditional and network meta-analysis suggested that the use of VCDs significantly decreased the risk of haematomas. Further, the newly developed VCDs used in the past decade in particular significantly reduced the rate of CAVE. Additionally, FemoSeal also reduced the risk of CAVE, indicating that newer VCDs with advanced design might improve the safety of VCDs. AngioSeal, which might be the best VCD among all the included VCDs, was associated with a reduced rate of both CAVE and haematomas compared with MC. However, these conclusions are still to be demonstrated by large-scale high-quality RCTs due to the inherent bias and heterogeneity of the RCTs included in our meta-analysis.

## Additional Information

**How to cite this article**: Jun, J. *et al.* Network Meta-analysis of Randomized Trials on the Safety of Vascular Closure Devices for Femoral Arterial Puncture Site Haemostasis. *Sci. Rep.* **5**, 13761; doi: 10.1038/srep13761 (2015).

## Supplementary Material

Supplementary Information

## Figures and Tables

**Table 1:** Risk of combined adverse vascular events of different vascular closure devices versus manual compression.

**Vascular closure devices**	**No. of studies**	**Total patients**	**M-H, Random**	**Heterogeneity**	**Test for overall effect**	
			**RR [95% CI]**	**Chi^2^/I^2^ value**	**Z value**	**P value**
AngioSeal	13	3264	0.69 [0.46, 1.03]	44.41/0.73	1.81	0.07
VasoSeal	7	1301	1.10 [0.75, 1.61]	22.68/0.74	0.46	0.64
ExoSeal	2	3416	1.45 [0.55, 3.84]	3.51/0.72	0.75	0.45
QuickSeal	2	539	1.27 [0.48, 3.37]	2.40/0.58	0.49	0.63
FemoSeal	2	4019	0.75 [0.60, 0.94]	0.00/0.00	2.46	0.01*
Perclose	9	2311	1.00 [0.65, 1.52]	14.89/0.46	0.02	0.99
StarClose	3	1132	0.63 [0.29, 1.37]	5.27/0.62	1.17	0.24

^*^Statistically significant.

**Table 2:**
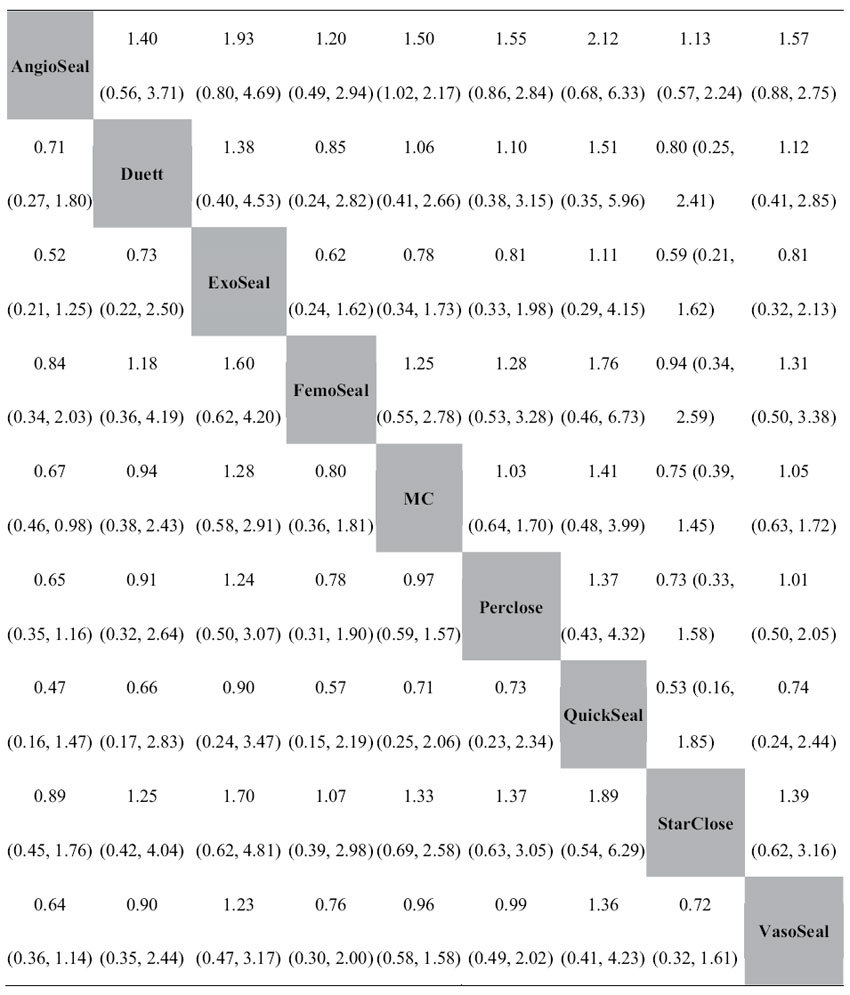
Network meta-analysis (consistency model) of the risk ratio and 95% confidence intervals of combined adverse vascular events associated with different vascular closure devices and manual compression.

MC, manual compression.

**Table 3:**
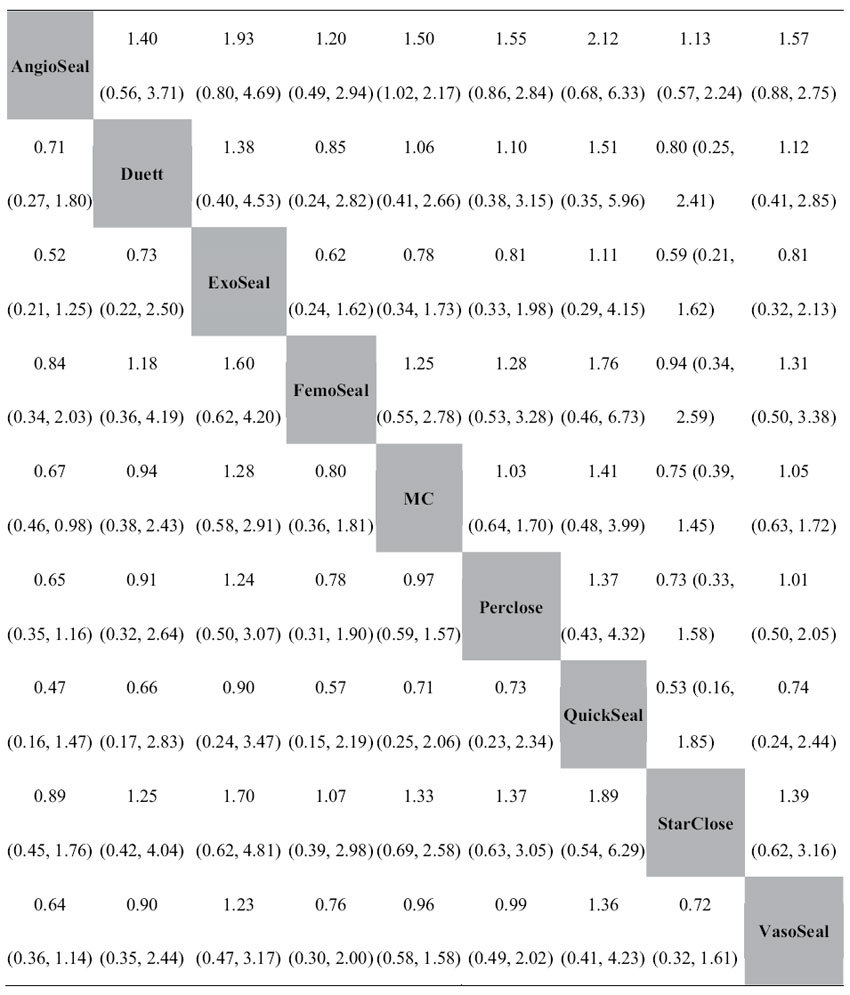
Network meta-analysis (consistency model) of the risk ratio and 95% confidence intervals of haematomas associated with different vascular closure devices and manual compression.

MC, manual compression.
