# Supplementary material for: Network Meta-analysis of Randomized Trials on the Safety of Vascular Closure Devices for Femoral Arterial Puncture Site Haemostasis
Source: Sci Rep. 2015 Sep 8;5:13761. doi: 10.1038/srep13761 (PMC4562233; doi:10.1038/srep13761)
Supplement: Supplementary Information [file srep13761-s1.pdf]

Supplementary Information

# Network Meta-analysis of Randomized Trials on the Safety of Vascular Closure Devices for Femoral Arterial Puncture Site Haemostasis

Jun Jiang MD, PhD, Junjie Zou MD, PhD, Hao Ma MD, PhD, Yuanyong Jiao MD, Hongyu Yang MD, Xiwei Zhang MD, Yi Miao MD, PhD

Supplementary Figure S1

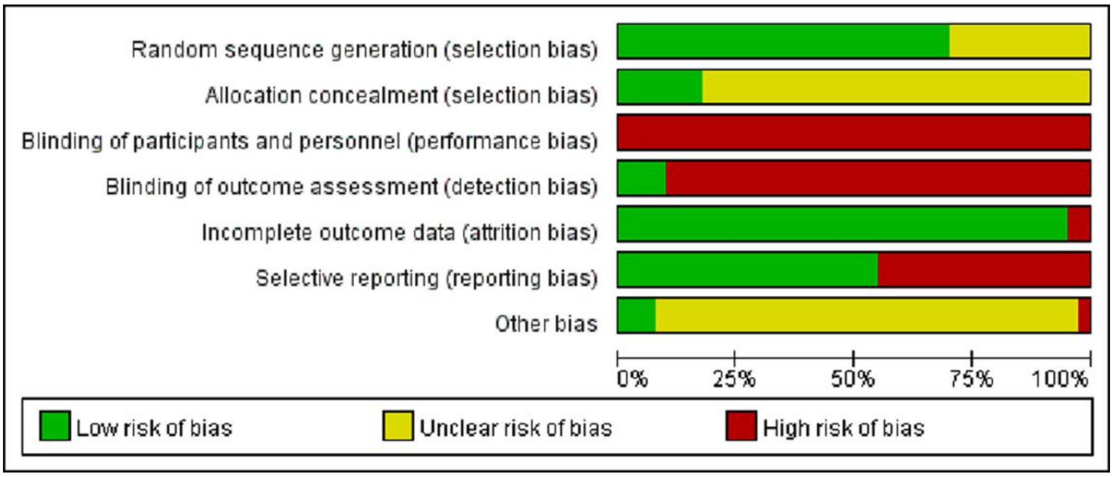


Risk of bias graph: the authors' conclusions about the risk of each bias item are presented as percentages across all the included studies.

Supplementary Figure S2

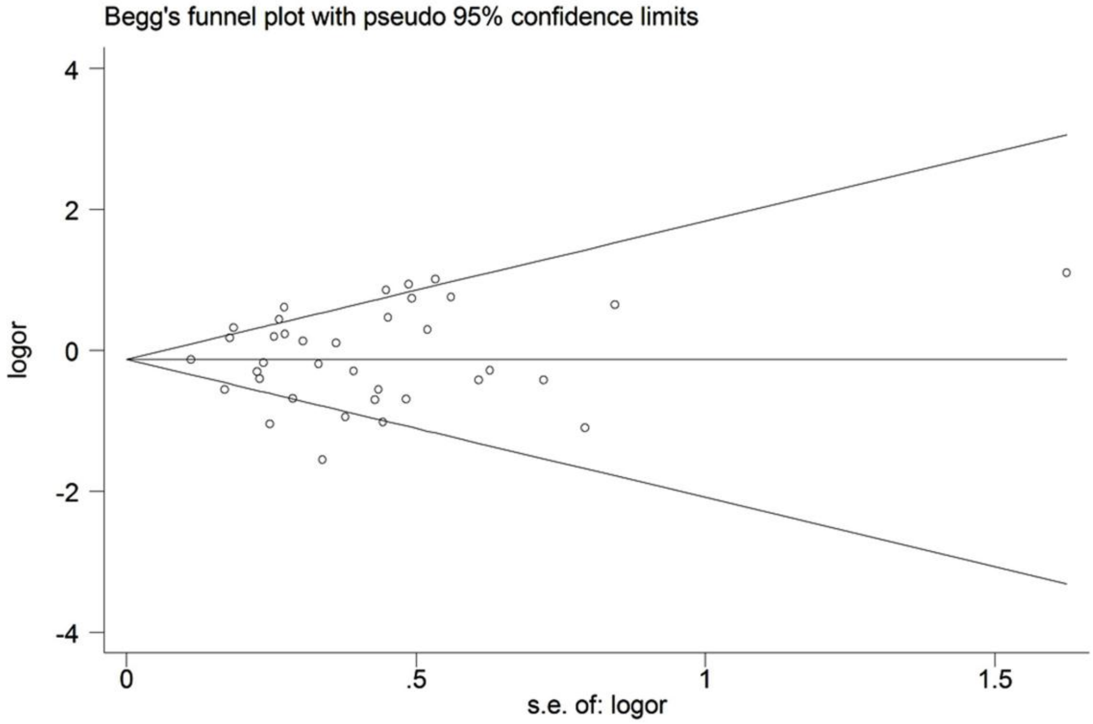


Funnel plot of all included trials for detection of publication bias (t = 0.35; *P* = 0.725).

Supplementary Figure S3

| Study or Subgroup | VCD Events | VCD Total | MC Events | MC Total | Weight | Risk Ratio M-H, Random, 95% CI | Year |
|---|---|---|---|---|---|---|---|
| **2.1.2 trials before 2005** | | | | | | | |
| Schräder 1992 | 23 | 50 | 40 | 50 | 6.7% | 0.57 [0.41, 0.80] | 1992 |
| Sanborn 1993 | 33 | 246 | 23 | 209 | 5.7% | 1.22 [0.74, 2.01] | 1993 |
| Camenzind 1994 | 36 | 62 | 26 | 62 | 6.5% | 1.38 [0.96, 1.99] | 1994 |
| Slaughter 1995 | 13 | 51 | 5 | 50 | 3.4% | 2.55 [0.98, 6.62] | 1995 |
| Kussmaul 1995 | 27 | 218 | 40 | 217 | 6.0% | 0.67 [0.43, 1.05] | 1995 |
| von Hoch 1995 | 31 | 154 | 20 | 155 | 5.6% | 1.56 [0.93, 2.61] | 1995 |
| Beyer-Enke 1996 | 30 | 48 | 25 | 48 | 6.6% | 1.20 [0.85, 1.70] | 1996 |
| Seidelin 1997 | 14 | 24 | 12 | 26 | 5.5% | 1.26 [0.74, 2.16] | 1997 |
| Gwechenberger 1997 | 3 | 33 | 4 | 29 | 2.0% | 0.66 [0.16, 2.70] | 1997 |
| Ward 1998 | 19 | 202 | 6 | 102 | 3.6% | 1.60 [0.66, 3.88] | 1998 |
| Silber 1998 | 22 | 74 | 27 | 76 | 5.9% | 0.84 [0.53, 1.33] | 1998 |
| Gerckens 1999 | 17 | 298 | 33 | 292 | 5.3% | 0.50 [0.29, 0.89] | 1999 |
| Baim 2000 | 12 | 251 | 6 | 264 | 3.3% | 2.10 [0.80, 5.52] | 2000 |
| Wetter 2000 | 9 | 50 | 12 | 50 | 4.2% | 0.75 [0.35, 1.62] | 2000 |
| Díaz De La Llera 2001 | 1 | 75 | 0 | 75 | 0.5% | 3.00 [0.12, 72.49] | 2001 |
| Rickli 2002 | 8 | 96 | 6 | 97 | 3.1% | 1.35 [0.49, 3.74] | 2002 |
| The STST 2002 | 14 | 392 | 4 | 238 | 2.8% | 2.13 [0.71, 6.38] | 2002 |
| Tron 2003 | 17 | 91 | 6 | 76 | 3.7% | 2.37 [0.98, 5.70] | 2003 |
| Chevalier 2003 | 20 | 306 | 57 | 306 | 5.8% | 0.35 [0.22, 0.57] | 2003 |
| Yadav 2003 | 45 | 240 | 16 | 158 | 5.5% | 1.85 [1.09, 3.16] | 2003 |
| Castañeda 2003 | 5 | 85 | 5 | 56 | 2.5% | 0.66 [0.20, 2.17] | 2003 |
| Starnes 2003 | 4 | 52 | 2 | 50 | 1.6% | 1.92 [0.37, 10.04] | 2003 |
| Reddy 2004 | 10 | 25 | 9 | 25 | 4.5% | 1.11 [0.55, 2.26] | 2004 |
| **Subtotal (95% CI)** | | **3123** | | **2711** | **100.0%** | **1.08 [0.85, 1.36]** | |
| Total events | 413 | | 384 | | | | |
| Heterogeneity: Tau² = 0.18; Chi² = 66.74, df = 22 (P < 0.00001); I² = 67% | | | | | | | |
| Test for overall effect: Z = 0.61 (P = 0.54) | | | | | | | |
| **2.1.3 trials after 2005** | | | | | | | |
| Legrand 2005 | 11 | 203 | 26 | 102 | 10.2% | 0.21 [0.11, 0.41] | 2005 |
| Ansel 2006 | 22 | 243 | 13 | 119 | 10.4% | 0.83 [0.43, 1.59] | 2006 |
| Hermiller 2006 | 10 | 184 | 10 | 91 | 8.3% | 0.49 [0.21, 1.15] | 2006 |
| Upponi 2007 | 2 | 50 | 6 | 50 | 3.7% | 0.33 [0.07, 1.57] | 2007 |
| Deuling 2008 | 32 | 300 | 14 | 150 | 11.0% | 1.14 [0.63, 2.08] | 2008 |
| Martin 2008 | 6 | 133 | 4 | 67 | 5.2% | 0.76 [0.22, 2.59] | 2008 |
| Wong 2009 | 22 | 267 | 4 | 134 | 6.5% | 2.76 [0.97, 7.85] | 2009 |
| Hermanides 2010 | 8 | 313 | 14 | 314 | 8.1% | 0.57 [0.24, 1.35] | 2010 |
| Machnik 2012 | 6 | 91 | 20 | 110 | 8.0% | 0.36 [0.15, 0.86] | 2012 |
| Schulz-Schüpke 2014 | 208 | 3015 | 119 | 1509 | 15.7% | 0.87 [0.70, 1.09] | 2014 |
| Holm 2014 | 32 | 501 | 43 | 500 | 13.0% | 0.74 [0.48, 1.15] | 2014 |
| **Subtotal (95% CI)** | | **5300** | | **3146** | **100.0%** | **0.67 [0.48, 0.94]** | |
| Total events | 359 | | 273 | | | | |
| Heterogeneity: Tau² = 0.18; Chi² = 28.79, df = 10 (P = 0.001); I² = 65% | | | | | | | |
| Test for overall effect: Z = 2.29 (P = 0.02) | | | | | | | |

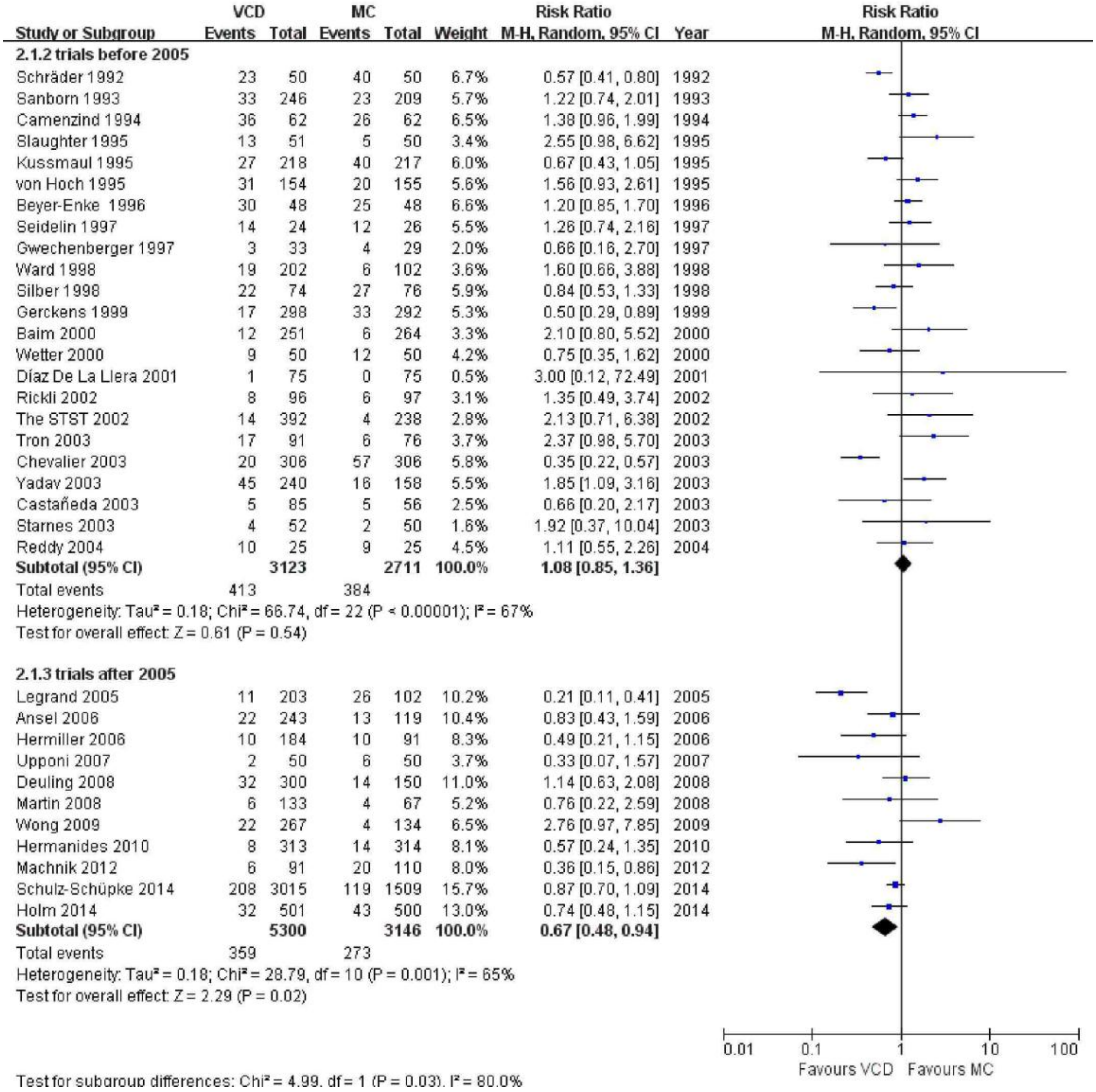


Test for subgroup differences: Chi² = 4.99, df = 1 (P = 0.03), I² = 80.0%

Subgroup analysis of included studies excluding two studies conducted in the East Asian population stratified by the year of publication accessing the risk of combined adverse vascular events of VCDs versus MC. VCD = vascular closure device, MC = manual compression, M-H = Mantel-Haenzel, CI = confidence interval.

Supplementary Figure S4

| Study or Subgroup | VCD Events | VCD Total | MC Events | MC Total | Weight | Risk Ratio M-H, Random, 95% CI | Year |
|---|---|---|---|---|---|---|---|
| **2.1.2 trials before 2005** | | | | | | | |
| Schräder 1992 | 23 | 50 | 40 | 50 | 6.7% | 0.57 [0.41, 0.80] | 1992 |
| Sanborn 1993 | 33 | 246 | 23 | 209 | 5.7% | 1.22 [0.74, 2.01] | 1993 |
| Camenzind 1994 | 36 | 62 | 26 | 62 | 6.5% | 1.38 [0.96, 1.99] | 1994 |
| Slaughter 1995 | 13 | 51 | 5 | 50 | 3.4% | 2.55 [0.98, 6.62] | 1995 |
| Kussmaul 1995 | 27 | 218 | 40 | 217 | 6.0% | 0.67 [0.43, 1.05] | 1995 |
| von Hoch 1995 | 31 | 154 | 20 | 155 | 5.6% | 1.56 [0.93, 2.61] | 1995 |
| Beyer-Enke 1996 | 30 | 48 | 25 | 48 | 6.6% | 1.20 [0.85, 1.70] | 1996 |
| Seidelin 1997 | 14 | 24 | 12 | 26 | 5.5% | 1.26 [0.74, 2.16] | 1997 |
| Gwechenberger 1997 | 3 | 33 | 4 | 29 | 2.0% | 0.66 [0.16, 2.70] | 1997 |
| Silber 1998 | 22 | 74 | 27 | 76 | 5.9% | 0.84 [0.53, 1.33] | 1998 |
| Ward 1998 | 19 | 202 | 6 | 102 | 3.7% | 1.60 [0.66, 3.88] | 1998 |
| Gerckens 1999 | 17 | 298 | 33 | 292 | 5.3% | 0.50 [0.29, 0.89] | 1999 |
| Wetter 2000 | 9 | 50 | 12 | 50 | 4.2% | 0.75 [0.35, 1.62] | 2000 |
| Baim 2000 | 12 | 251 | 6 | 264 | 3.3% | 2.10 [0.80, 5.52] | 2000 |
| The STST 2002 | 14 | 392 | 4 | 238 | 2.9% | 2.13 [0.71, 6.38] | 2002 |
| Rickli 2002 | 8 | 96 | 6 | 97 | 3.1% | 1.35 [0.49, 3.74] | 2002 |
| Starnes 2003 | 4 | 52 | 2 | 50 | 1.6% | 1.92 [0.37, 10.04] | 2003 |
| Chevalier 2003 | 20 | 306 | 57 | 306 | 5.8% | 0.35 [0.22, 0.57] | 2003 |
| Tron 2003 | 17 | 91 | 6 | 76 | 3.7% | 2.37 [0.98, 5.70] | 2003 |
| Yadav 2003 | 45 | 240 | 16 | 158 | 5.5% | 1.85 [1.09, 3.16] | 2003 |
| Castañeda 2003 | 5 | 85 | 5 | 56 | 2.6% | 0.66 [0.20, 2.17] | 2003 |
| Reddy 2004 | 10 | 25 | 9 | 25 | 4.5% | 1.11 [0.55, 2.26] | 2004 |
| **Subtotal (95% CI)** | | **3048** | | **2636** | **100.0%** | **1.07 [0.85, 1.35]** | |
| Total events | 412 | | 384 | | | | |
| Heterogeneity: Tau² = 0.19; Chi² = 66.27, df = 21 (P < 0.00001); I² = 68% | | | | | | | |
| Test for overall effect: Z = 0.57 (P = 0.57) | | | | | | | |
| **2.1.3 trials after 2005** | | | | | | | |
| Legrand 2005 | 11 | 203 | 26 | 102 | 10.2% | 0.21 [0.11, 0.41] | 2005 |
| Hermiller 2006 | 10 | 184 | 10 | 91 | 8.3% | 0.49 [0.21, 1.15] | 2006 |
| Ansel 2006 | 22 | 243 | 13 | 119 | 10.4% | 0.83 [0.43, 1.59] | 2006 |
| Upponi 2007 | 2 | 50 | 6 | 50 | 3.7% | 0.33 [0.07, 1.57] | 2007 |
| Martin 2008 | 6 | 133 | 4 | 67 | 5.2% | 0.76 [0.22, 2.59] | 2008 |
| Deuling 2008 | 32 | 300 | 14 | 150 | 11.0% | 1.14 [0.63, 2.08] | 2008 |
| Wong 2009 | 22 | 267 | 4 | 134 | 6.5% | 2.76 [0.97, 7.85] | 2009 |
| Hermanides 2010 | 8 | 313 | 14 | 314 | 8.1% | 0.57 [0.24, 1.35] | 2010 |
| Machnik 2012 | 6 | 91 | 20 | 110 | 8.0% | 0.36 [0.15, 0.86] | 2012 |
| Holm 2014 | 32 | 501 | 43 | 500 | 13.0% | 0.74 [0.48, 1.15] | 2014 |
| Schulz-Schüpke 2014 | 208 | 3015 | 119 | 1509 | 15.7% | 0.87 [0.70, 1.09] | 2014 |
| **Subtotal (95% CI)** | | **5300** | | **3146** | **100.0%** | **0.67 [0.48, 0.94]** | |
| Total events | 359 | | 273 | | | | |
| Heterogeneity: Tau² = 0.18; Chi² = 28.79, df = 10 (P = 0.001); I² = 65% | | | | | | | |
| Test for overall effect: Z = 2.29 (P = 0.02) | | | | | | | |

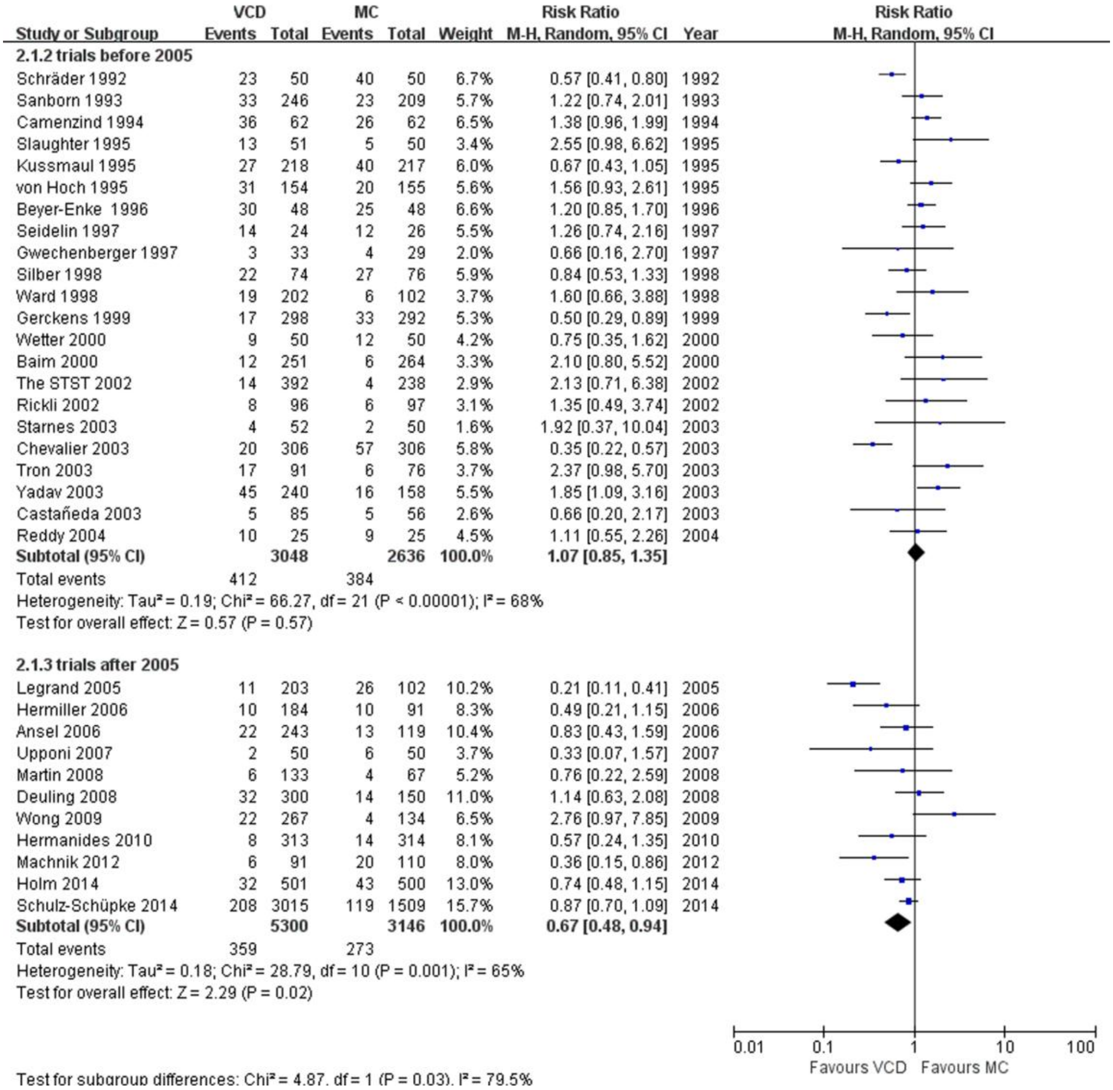


Test for subgroup differences: Chi² = 4.87, df = 1 (P = 0.03), I² = 79.5%

Subgroup analysis of included studies excluding two non-English language studies stratified by the year of publication accessing the risk of combined adverse vascular events of VCDs versus MC. VCD = vascular closure device, MC = manual compression, M-H = Mantel-Haenzel, CI = confidence interval.

Supplementary Figure S5

| Study or Subgroup | VCD Events | VCD Total | MC Events | MC Total | Weight | Risk Ratio M-H, Fixed, 95% CI | Year |
|---|---|---|---|---|---|---|---|
| Schräder 1992 | 23 | 50 | 34 | 50 | 6.7% | 0.68 [0.47, 0.97] | 1992 |
| Sanborn 1993 | 20 | 246 | 17 | 209 | 3.6% | 1.00 [0.54, 1.86] | 1993 |
| Camenzind 1994 | 30 | 62 | 22 | 62 | 4.3% | 1.36 [0.89, 2.08] | 1994 |
| Kussmaul 1995 | 5 | 218 | 12 | 217 | 2.4% | 0.41 [0.15, 1.16] | 1995 |
| Slaughter 1995 | 9 | 51 | 5 | 50 | 1.0% | 1.76 [0.64, 4.90] | 1995 |
| Beyer-Enke 1996 | 21 | 48 | 22 | 48 | 4.3% | 0.95 [0.61, 1.49] | 1996 |
| Seidelin 1997 | 12 | 24 | 12 | 26 | 2.3% | 1.08 [0.61, 1.93] | 1997 |
| Gwechenberger 1997 | 1 | 33 | 2 | 29 | 0.4% | 0.44 [0.04, 4.60] | 1997 |
| Ward 1998 | 8 | 202 | 4 | 102 | 1.0% | 1.01 [0.31, 3.27] | 1998 |
| Silber 1998 | 22 | 74 | 27 | 76 | 5.2% | 0.84 [0.53, 1.33] | 1998 |
| Gerckens 1999 | 11 | 298 | 21 | 292 | 4.2% | 0.51 [0.25, 1.05] | 1999 |
| Baim 2000 | 6 | 251 | 3 | 264 | 0.6% | 2.10 [0.53, 8.32] | 2000 |
| Wetter 2000 | 8 | 50 | 11 | 50 | 2.2% | 0.73 [0.32, 1.65] | 2000 |
| Díaz De La Llera 2001 | 1 | 75 | 0 | 75 | 0.1% | 3.00 [0.12, 72.49] | 2001 |
| Rickli 2002 | 7 | 96 | 5 | 97 | 1.0% | 1.41 [0.47, 4.30] | 2002 |
| Chevalier 2003 | 10 | 306 | 11 | 306 | 2.2% | 0.91 [0.39, 2.11] | 2003 |
| Tron 2003 | 5 | 91 | 2 | 76 | 0.4% | 2.09 [0.42, 10.46] | 2003 |
| Castañeda 2003 | 5 | 85 | 5 | 56 | 1.2% | 0.66 [0.20, 2.17] | 2003 |
| Yadav 2003 | 37 | 240 | 13 | 158 | 3.1% | 1.87 [1.03, 3.41] | 2003 |
| Reddy 2004 | 9 | 25 | 9 | 25 | 1.8% | 1.00 [0.48, 2.09] | 2004 |
| Legrand 2005 | 11 | 203 | 24 | 102 | 6.3% | 0.23 [0.12, 0.45] | 2005 |
| Hermiller 2006 | 8 | 184 | 7 | 91 | 1.8% | 0.57 [0.21, 1.51] | 2006 |
| Upponi 2007 | 2 | 50 | 6 | 50 | 1.2% | 0.33 [0.07, 1.57] | 2007 |
| Deuling 2008 | 32 | 300 | 14 | 150 | 3.7% | 1.14 [0.63, 2.08] | 2008 |
| Wong 2009 | 6 | 267 | 1 | 134 | 0.3% | 3.01 [0.37, 24.76] | 2009 |
| Hermanides 2010 | 5 | 313 | 11 | 314 | 2.2% | 0.46 [0.16, 1.30] | 2010 |
| Machnik 2012 | 6 | 91 | 13 | 110 | 2.3% | 0.56 [0.22, 1.41] | 2012 |
| Schulz-Schüpke 2014 | 145 | 3015 | 102 | 1509 | 26.8% | 0.71 [0.56, 0.91] | 2014 |
| Holm 2014 | 29 | 501 | 38 | 500 | 7.5% | 0.76 [0.48, 1.22] | 2014 |
| **Total (95% CI)** | | **7449** | | **5228** | **100.0%** | **0.82 [0.73, 0.92]** | |
| Total events | 494 | | 453 | | | | |

Heterogeneity: Chi² = 48.31, df = 28 (P = 0.010); I² = 42%

Test for overall effect: Z = 3.37 (P = 0.0008)

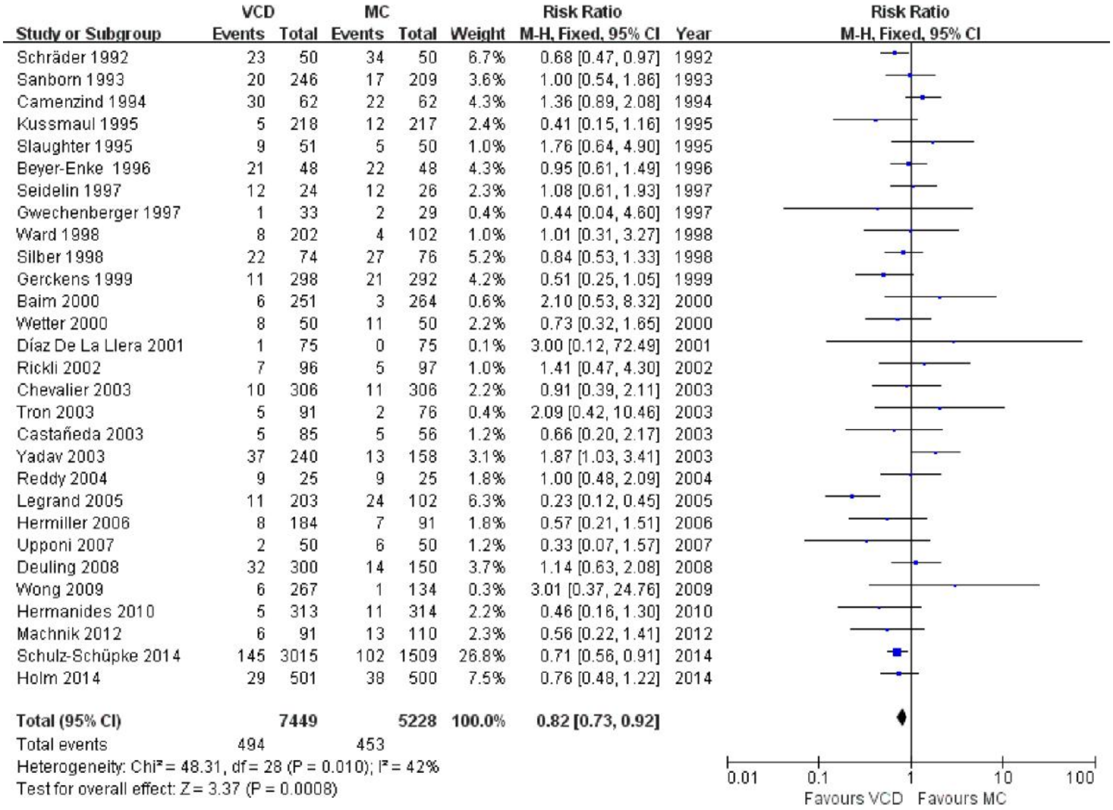


Risk of haematomas associated with all VCDs versus MC of the included studies excluding two studies conducted in the East Asian population. VCD = vascular closure device, MC = manual compression, M-H = Mantel-Haenzel, CI = confidence interval.

Supplementary Figure S6

| Study or Subgroup | VCD Events | VCD Total | MC Events | MC Total | Weight | Risk Ratio M-H, Fixed, 95% CI | Year |
|---|---|---|---|---|---|---|---|
| Schräder 1992 | 23 | 50 | 34 | 50 | 6.6% | 0.68 [0.47, 0.97] | 1992 |
| Sanborn 1993 | 20 | 246 | 17 | 209 | 3.6% | 1.00 [0.54, 1.86] | 1993 |
| Camenzind 1994 | 30 | 62 | 22 | 62 | 4.3% | 1.36 [0.89, 2.08] | 1994 |
| Kussmaul 1995 | 5 | 218 | 12 | 217 | 2.3% | 0.41 [0.15, 1.16] | 1995 |
| Slaughter 1995 | 9 | 51 | 5 | 50 | 1.0% | 1.76 [0.64, 4.90] | 1995 |
| Beyer-Enke 1996 | 21 | 48 | 22 | 48 | 4.3% | 0.95 [0.61, 1.49] | 1996 |
| Seidelin 1997 | 12 | 24 | 12 | 26 | 2.2% | 1.08 [0.61, 1.93] | 1997 |
| Gwechenberger 1997 | 1 | 33 | 2 | 29 | 0.4% | 0.44 [0.04, 4.60] | 1997 |
| Ward 1998 | 8 | 202 | 4 | 102 | 1.0% | 1.01 [0.31, 3.27] | 1998 |
| Silber 1998 | 22 | 74 | 27 | 76 | 5.2% | 0.84 [0.53, 1.33] | 1998 |
| Gerckens 1999 | 11 | 298 | 21 | 292 | 4.1% | 0.51 [0.25, 1.05] | 1999 |
| Wetter 2000 | 8 | 50 | 11 | 50 | 2.1% | 0.73 [0.32, 1.65] | 2000 |
| Noguchi 2000 | 4 | 30 | 10 | 30 | 1.9% | 0.40 [0.14, 1.14] | 2000 |
| Baim 2000 | 6 | 251 | 3 | 264 | 0.6% | 2.10 [0.53, 8.32] | 2000 |
| Rickli 2002 | 7 | 96 | 5 | 97 | 1.0% | 1.41 [0.47, 4.30] | 2002 |
| Tron 2003 | 5 | 91 | 2 | 76 | 0.4% | 2.09 [0.42, 10.46] | 2003 |
| Yadav 2003 | 37 | 240 | 13 | 158 | 3.0% | 1.87 [1.03, 3.41] | 2003 |
| Castañeda 2003 | 5 | 85 | 5 | 56 | 1.2% | 0.66 [0.20, 2.17] | 2003 |
| Chevalier 2003 | 10 | 306 | 11 | 306 | 2.1% | 0.91 [0.39, 2.11] | 2003 |
| Reddy 2004 | 9 | 25 | 9 | 25 | 1.7% | 1.00 [0.48, 2.09] | 2004 |
| Legrand 2005 | 11 | 203 | 24 | 102 | 6.2% | 0.23 [0.12, 0.45] | 2005 |
| Hermiller 2006 | 8 | 184 | 7 | 91 | 1.8% | 0.57 [0.21, 1.51] | 2006 |
| Upponi 2007 | 2 | 50 | 6 | 50 | 1.2% | 0.33 [0.07, 1.57] | 2007 |
| Deuling 2008 | 32 | 300 | 14 | 150 | 3.6% | 1.14 [0.63, 2.08] | 2008 |
| Wong 2009 | 6 | 267 | 1 | 134 | 0.3% | 3.01 [0.37, 24.76] | 2009 |
| Hermanides 2010 | 5 | 313 | 11 | 314 | 2.1% | 0.46 [0.16, 1.30] | 2010 |
| Machnik 2012 | 6 | 91 | 13 | 110 | 2.3% | 0.56 [0.22, 1.41] | 2012 |
| Schulz-Schüpke 2014 | 145 | 3015 | 102 | 1509 | 26.3% | 0.71 [0.56, 0.91] | 2014 |
| Holm 2014 | 29 | 501 | 38 | 500 | 7.4% | 0.76 [0.48, 1.22] | 2014 |
| **Total (95% CI)** | | **7404** | | **5183** | **100.0%** | **0.81 [0.72, 0.91]** | |
| Total events | 497 | | 463 | | | | |

Heterogeneity: Chi² = 49.52, df = 28 (P = 0.007); I² = 43%

Test for overall effect: Z = 3.60 (P = 0.0003)

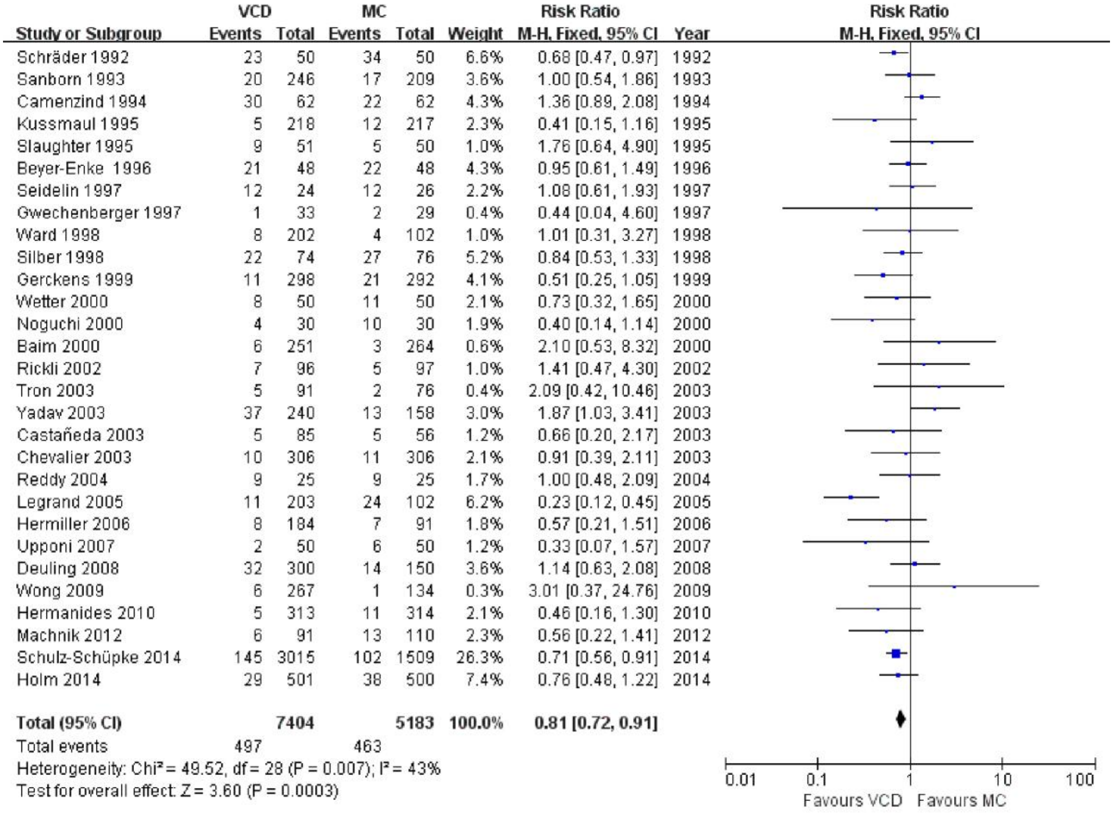


Risk of haematomas associated with all VCDs versus MC of included studies excluding two non-English language studies. VCD = vascular closure device, MC = manual compression, M-H = Mantel-Haenzel, CI = confidence interval.

Supplementary Table S1. Characteristics of the included studies

| Studies | Year of publication | VCD, No. of patients | Control, No. of patients | Country | Funding | Follow-up (d) | Jadad score |
|---|---|---|---|---|---|---|---|
| Schräder | 1992 | VasoSeal, 50 | MC, 50 | Germany | NR | 42 | 3 |
| Sanborn | 1993 | VasoSeal, 246 | MC, 209 | USA | Datascope Corp. | 30 | 3 |
| Camenzind | 1994 | VasoSeal, 62 | MC, 62 | Switzerland | NR | 1 | 3 |
| von Hoch | 1995 | VasoSeal, 154 | MC, 155 | German | NR | 7 | 2 |
| Slaughter | 1995 | VasoSeal, 51 | MC, 50 | Canada | NR | 35 | 3 |
| Kussmaul | 1995 | AngioSeal, 218 | MC, 217 | USA | NR | 60 | 3 |
| Beyer-Enke | 1996 | AngioSeal, 48 | MC, 48 | Germany | NR | 84 | 2 |
| Seidelin | 1997 | AngioSeal, 24 | MC, 26 | Canada | Sherwood Davis & Geck | 7 | 3 |
| Gwechenberger | 1997 | VasoSeal, 33 | MC, 29 | Austria | NR | 7 | 2 |
| Ward | 1998 | AngioSeal, 202 | MC, 102 | USA | Sherwood Davis & Geck | 30 | 2 |
| Silber | 1998 | VasoSeal, 74 | MC, 76 | Germany | NR | 1 | 2 |
| Gerckens | 1999 | Perclose (Techstar or Prostar), 298 | MC, 292 | Germany | NR | 15 | 3 |
| Wetter | 2000 | Perclose (Techstar), 50 | MC, 50 | Switzerland | Philips Medical Systems, Inc. | In hospital | 3 |
| Noguchi | 2000 | Perclose (Prostar Plus), 30 | MC, 30 | Japan | NR | 1 | 2 |
| Baim | 2000 | Perclose (Prostar Plus), 251 | MC, 264 | USA | NR | 30 | 2 |
| Díaz De La Llera | 2001 | AngioSeal, 75 | MC, 75 | Sevilla | NR | 70 | 3 |

| | | | | | | | |
|---|---|---|---|---|---|---|---|
| The STST | 2002 | Duett, 392 | MC, 238 | USA | NR | 30 | 3 |
| Shammas | 2002 | AngioSeal, 77 | VasoSeal, 73 | USA | Datascope Corp. | 30 | 2 |
| Rickli | 2002 | Perclose(Techstar XL), 96 | MC, 97 | Switzerland | NR | 90 | 2 |
| Michalis | 2002 | Angioseal, 290; VasoSeal, 280 | Duett, 281 | Greece | NR | In hospital | 2 |
| Yadav | 2003 | QuickSeal, 240 | MC, 158 | USA | SUB-Q Inc. | 30 | 2 |
| Tron | 2003 | Perclose, 91 | MC, 76 | France | NR | In hospital | 2 |
| Starnes | 2003 | Perclose (Techstar，Prostar XL), 52 | MC, 50 | USA | NR | 405 | 2 |
| Chevalier | 2003 | AngioSeal, 306 | MC, 306 | France | Daig Division | 7 | 3 |
| Castañeda | 2003 | QuickSeal, 85 | MC, 56 | USA | NR | 30 | 3 |
| Reddy | 2004 | AngioSeal, 25 | MC, 25 | USA | NR | 7 | 2 |
| Legrand | 2005 | AngioSeal, 100; Bio-DISC, 103 | MC, 102 | Belgium | NR | 1 | 3 |
| Hermiller | 2006 | StarClose, 184 | MC, 91 | USA | Abbott Vascular | In hospital | 2 |
| Ansel | 2006 | EVS,243 | MC, 119 | USA | Angiolink Co. | 30 | 2 |
| Upponi | 2007 | Angioseal, 50 | MC, 50 | UK | NR | 7 | 2 |
| Rastan | 2008 | AngioSeal,285; StarClose, 286 | D-Stat Dry, 281 | Switzerland | NR | 1 | 3 |
| Martin | 2008 | AngioSeal,70; Perclose, 63 | MC, 67 | USA | NR | In hospital | 2 |
| Jensen | 2008 | AngioSeal,22; Perclose, 22 | FemoStop, 24 | Sweden | NR | 30 | 2 |

| | | | | | | | |
|---|---|---|---|---|---|---|---|
| Deuling | 2008 | AngioSeal,150; StarClose, 150 | MC, 150 | Netherlands | NR | 1 | 2 |
| Wong | 2009 | ExoSeal, 267 | MC, 134 | USA | Cordis Corp. | 30 | 3 |
| Sun | 2009 | StarClose,286; Perclose (PROGLIDE),183; Boomerang, 178 | MC, 271 | China | NR | In hospital | 2 |
| Hermanides | 2010 | AngioSeal, 313 | MC, 314 | Netherlands | partly from St. Jude Medical | In hospital | 3 |
| Machnik | 2012 | AngioSeal, 91 | MC, 110 | Poland | NR | In hospital | 2 |
| Schulz-Schüpke | 2014 | FemoSeal, 1509; ExoSeal, 1506 | MC, 1509 | Germany | Deutsches Herzzentrum | 30 | 3 |
| Holm | 2014 | FemoSeal, 501 | MC, 500 | Denmark | NR | In hospital | 3 |

NR = not reported, VCD = vascular closure device, MC, manual compression.

Supplementary Table S2. Node-splitting analysis of direct and indirect comparison of different vascular closure devices and manual compression

| Name | Direct Effect | Indirect Effect | Overall | P-Value |
|---|---|---|---|---|
| AngioSeal , Duett | -0.17<br>(-1.48, 1.10) | 1.28<br>(-0.28, 2.96) | 0.34<br>(-0.59, 1.31) | 0.14 |
| AngioSeal , MC | 0.40<br>(-0.03, 0.82) | 0.41<br>(-0.34, 1.12) | 0.40<br>(0.02, 0.78) | 0.99 |
| AngioSeal , Perclose | 0.20<br>(-1.19, 1.55) | 0.49<br>(-0.17, 1.14) | 0.44<br>(-0.15, 1.04) | 0.70 |
| AngioSeal , StarClose | 0.51<br>(-0.48, 1.49) | -0.41<br>(-1.38, 0.55) | 0.12<br>(-0.56, 0.81) | 0.18 |
| AngioSeal , VasoSeal | 0.35<br>(-0.65, 1.33) | 0.44<br>(-0.30, 1.16) | 0.45<br>(-0.13, 1.01) | 0.85 |
| Duett , MC | -0.82<br>(-2.46, 0.73) | 0.56<br>(-0.60, 1.76) | 0.06<br>(-0.89, 0.98) | 0.15 |
| Duett , VasoSeal | 0.41<br>(-0.87, 1.75) | -0.02<br>(-1.22, 1.12) | 0.11<br>(-0.89, 1.05) | 0.60 |
| ExoSeal , FemoSeal | -0.30<br>(-1.53, 0.95) | -0.58<br>(-2.13, 0.93) | -0.47<br>(-1.44, 0.48) | 0.76 |
| ExoSeal , MC | -0.39<br>(-1.36, 0.53) | 0.16<br>(-1.20, 1.57) | -0.25<br>(-1.07, 0.55) | 0.50 |
| ExoSeal , Perclose | 0.48<br>(-1.78, 2.95) | -0.35<br>(-1.37, 0.62) | -0.22<br>(-1.12, 0.69) | 0.50 |
| MC, Perclose | 0.02<br>(-0.49, 0.55) | -0.09<br>(-1.69, 1.48) | 0.03<br>(-0.45, 0.53) | 0.89 |
| MC, StarClose | -0.45<br>(-1.20, 0.29) | 0.54<br>(-0.94, 2.18) | -0.29<br>(-0.95, 0.37) | 0.25 |
| MC, VasoSeal | 0.04<br>(-0.56, 0.64) | 0.06<br>(-0.95, 1.12) | 0.04<br>(-0.46, 0.54) | 0.97 |
| Perclose , StarClose | -1.04<br>(-2.64, 0.47) | -0.05<br>(-0.92, 0.83) | -0.32<br>(-1.12, 0.46) | 0.26 |

MC, manual compression.

Supplementary Table S3. Network meta-analysis (consistency model) of the risk ratio and 95% confidence intervals of haematomas associated with different vascular closure devices and manual compression of included studies excluding two studies conducted in the East Asian population

| | | | | | | | |
|---|---|---|---|---|---|---|---|
| **AngioSeal** | 1.62 (0.68, 4.56) | 1.20 (0.54, 2.82) | 1.67 (1.13, 2.50) | 1.47 (0.75, 3.15) | 2.44 (0.84, 6.76) | 1.45 (0.64, 3.11) | 1.68 (0.91, 3.04) |
| 0.62 (0.22, 1.46) | **ExoSeal** | 0.74 (0.28, 1.75) | 1.03 (0.40, 2.23) | 0.90 (0.30, 2.50) | 1.51 (0.36, 5.00) | 0.89 (0.25, 2.60) | 1.03 (0.35, 2.56) |
| 0.84 (0.36, 1.87) | 1.36 (0.57, 3.61) | **FemoSeal** | 1.41 (0.66, 2.81) | 1.22 (0.49, 3.21) | 2.07 (0.57, 6.46) | 1.22 (0.39, 3.43) | 1.40 (0.55, 3.23) |
| 0.60 (0.40, 0.88) | 0.97 (0.45, 2.51) | 0.71 (0.36, 1.52) | **MC** | 0.88 (0.49, 1.73) | 1.46 (0.55, 3.76) | 0.86 (0.39, 1.85) | 1.00 (0.59, 1.66) |
| 0.68 (0.32, 1.34) | 1.12 (0.40, 3.29) | 0.82 (0.31, 2.06) | 1.14 (0.58, 2.05) | **Perclose** | 1.65 (0.49, 4.94) | 0.98 (0.34, 2.56) | 1.14 (0.48, 2.43) |
| 0.41 (0.15, 1.19) | 0.66 (0.20, 2.81) | 0.48 (0.15, 1.76) | 0.69 (0.27, 1.83) | 0.61 (0.20, 2.05) | **QuickSeal** | 0.59 (0.18, 2.07) | 0.69 (0.23, 2.08) |
| 0.69 (0.32, 1.55) | 1.13 (0.38, 4.06) | 0.82 (0.29, 2.53) | 1.16 (0.54, 2.56) | 1.02 (0.39, 2.91) | 1.69 (0.48, 5.67) | **StarClose** | 1.14 (0.48, 2.92) |
| 0.60 (0.33, 1.10) | 0.97 (0.39, 2.88) | 0.71 (0.31, 1.82) | 1.00 (0.60, 1.68) | 0.88 (0.41, 2.10) | 1.45 (0.48, 4.27) | 0.88 (0.34, 2.10) | **VasoSeal** |

MC, manual compression.

Supplementary Table S4. Network meta-analysis (consistency model) of the risk ratio and 95% confidence intervals of haematomas associated with different vascular closure devices and manual compression of included studies excluding two non-English language studies

| | | | | | | | |
|---|---|---|---|---|---|---|---|
| **AngioSeal** | 1.68 (0.71, 4.80) | 1.24 (0.56, 2.93) | 1.73 (1.16, 2.56) | 1.31 (0.68, 2.60) | 2.48 (0.87, 6.63) | 1.44 (0.67, 3.26) | 1.71 (0.95, 3.19) |
| 0.60 (0.21, 1.40) | **ExoSeal** | 0.74 (0.27, 1.70) | 1.03 (0.39, 2.20) | 0.78 (0.25, 2.02) | 1.46 (0.36, 4.86) | 0.86 (0.25, 2.56) | 1.02 (0.35, 2.53) |
| 0.81 (0.34, 1.78) | 1.36 (0.59, 3.69) | **FemoSeal** | 1.40 (0.64, 2.77) | 1.06 (0.41, 2.67) | 2.02 (0.57, 6.21) | 1.16 (0.40, 3.37) | 1.39 (0.55, 3.27) |
| 0.58 (0.39, 0.87) | 0.97 (0.45, 2.56) | 0.71 (0.36, 1.56) | **MC** | 0.75 (0.43, 1.38) | 1.45 (0.55, 3.56) | 0.84 (0.39, 1.88) | 0.99 (0.60, 1.66) |
| 0.77 (0.38, 1.48) | 1.29 (0.49, 4.00) | 0.95 (0.37, 2.45) | 1.33 (0.73, 2.33) | **Perclose** | 1.91 (0.59, 5.47) | 1.11 (0.41, 2.96) | 1.31 (0.60, 2.80) |
| 0.40 (0.15, 1.15) | 0.68 (0.21, 2.75) | 0.49 (0.16, 1.75) | 0.69 (0.28, 1.83) | 0.52 (0.18, 1.68) | **QuickSeal** | 0.58 (0.18, 2.06) | 0.69 (0.24, 2.09) |
| 0.69 (0.31, 1.49) | 1.17 (0.39, 4.02) | 0.86 (0.30, 2.49) | 1.19 (0.53, 2.55) | 0.90 (0.34, 2.45) | 1.71 (0.49, 5.66) | **StarClose** | 1.18 (0.46, 2.96) |
| 0.58 (0.31, 1.06) | 0.98 (0.40, 2.90) | 0.72 (0.31, 1.81) | 1.01 (0.60, 1.67) | 0.76 (0.36, 1.67) | 1.45 (0.48, 4.11) | 0.85 (0.34, 2.17) | **VasoSeal** |

MC, manual compression.
